# Microstructure and Mechanical Properties of Stainless Steel/6082 Aluminum Alloy Heterogeneous Laser Welded Joint

**DOI:** 10.3390/ma16216958

**Published:** 2023-10-30

**Authors:** Lei Kang, Xin Li, Jing Chen, Yu Zhang, Ting Wang

**Affiliations:** 1 State Key Laboratory of Automobile Materials, Jilin University, Changchun 130022, China; kanglei0618@163.com (L.K.); li_xin@jlu.edu.cn (X.L.); cj1540307503@163.com (J.C.); 18356472302@163.com (Y.Z.); 2 Jilin China Department of Materials Science and Engineering, Jilin University, Changchun 130022, China

**Keywords:** laser welding, steel/aluminum welding, intermetallic compounds, microstructure

## Abstract

The microstructures and mechanical properties of laser penetration welded joints of overlap steel-on-aluminum were investigated. The structure of the intermetallic compound layer without interlayer consists of FeAl and FeAl_3_ phases. After the Ni-foil was added, the thickness of the intermetallic compound layer and the content of the brittle and hard Al-Fe phase decreased significantly, and some new phases of Al_0.9_Ni_1.1_ and FeNi were formed. It was found that the Ni interlayer enhanced the tensile property of the joint by about 40% and decreased the microhardness of the intermetallic compounds, which is attributed to the improvement of the toughness of the welded joint made by the Ni interlayer. It is an effective way to improve the mechanical properties of the laser welding joint by adding a nickel interlayer to improve the metallurgical reaction.

## 1. Introduction

With the increasing demand for lightweight bodies in rail cars, lightweight materials, such as aluminum honeycomb panels, have been used in large quantities in interior body parts to reduce the weight of the body. However, the weight of the body itself still plays a major role in the process. Since entering the 21st century, researchers have focused their studies on the development of steel/aluminum heterogeneous metal hybrid structure bodies [1]. The use of steel–aluminum connection technology to achieve a lightweight car body has been widely used in automotive production, mainly using the mechanical connection method connection to achieve steel–aluminum connection. But the mechanical connection will increase the body mass and easily deform, so the welding method to realize the connection of steel and aluminum is a better production process to achieve a lightweight body. Laser welding has the characteristics of high energy density, small welding deformation and stress, fast welding speed, high production efficiency, easy to realize automation, and is a better welding method that can realize the connection between steel and aluminum [2,3,4]. The joining methods for steel–aluminum composite structures are currently studied as laser brazing [5,6,7], laser deep penetration welding [8,9], laser arc hybrid welding [10,11], and dual-beam laser small-hole welding [12]. However, due to the large differences in physical and chemical properties and atomic structure of steel and aluminum alloys, various brittle Al-Fe intermetallic compounds (IMCs), Fe_3_Al, FeAl_2_, Fe_2_Al_5_ and FeAl_3,_ are prone to appear in welded joints [13], and these intermetallic compounds will affect the performance of welded joints. With laser welding–brazing of aluminum to steel, the thickness of the brittle reaction layer can be limited to a thickness of just a few microns, which can significantly enhance the mechanical properties of the joint [14,15]. However, with the direct welding of steel and aluminum, the generation of brittle and hard steel and aluminum compounds is inevitable, which will seriously affect the strength of the joint. Therefore, adding a transition layer in the middle of the steel and aluminum is considered to improve the performance of the steel and aluminum joints. Zhang et al. [16] used 5251 automotive aluminum alloy and third-generation TG-1 steel as the research object; when aluminum was welded to steel, the most important problem was found to be that the strength of intermetallic compounds in the interface between steel and aluminum is generally low, which may directly lead to a decrease in the mechanical properties of the joints, and embrittlement will occur. Some scholars have proposed a steel–aluminum laser lap joint structure to inhibit the formation of IMC by controlling the penetration of steel in aluminum [17]. When steel and aluminum are welded directly, the generation of brittle and hard steel and aluminum compounds is inevitable, which will seriously affect the strength of the joint. Since the thermal conductivity of nickel is 90.7 W·m^−1^·K^−1^, which is in the middle of the thermal conductivity of aluminum alloy and stainless steel of 174 W·m^−1^·K^−1^ and 16.3 W·m^−1^·K^−1^, respectively, the thermal conductivity of the added nickel interlayer does not differ much from that of no interlayer, so nickel can be used as a transition metal for steel–aluminum fusion. Chen et al. [18] found that, by placing a layer of nickel foil interlayer made of 99.9% nickel between the laser welded plates of 201 stainless steel and 5052 aluminum alloy with a CO_2_ laser-focused spot diameter of 0.2 mm, compared to the joints without nickel foil, the fracture area of the IMCs of the joints with added nickel foil was significantly reduced. Xu et al. [19] added different thicknesses of nickel interlayer in SUS202 stainless steel and 2A12 aluminum alloy and studied the effect of different thicknesses of nickel interlayer on the mechanical properties and microstructure of welded joints. The results showed that, due to the addition of nickel, the melting of steel to aluminum was promoted, and the tensile strength of the welded joints was up to 103 N/mm when the nickel interlayer was 20 μm, and is improved by the addition of a nickel intermediate layer. Overall, the welded joints with nickel interlayer had higher tensile shear resistance than those without nickel interlayer. However, previous studies have shown that, for improving the mechanical properties of the joint, the addition of additional nickel elements is beneficial, but it is far less decisive than the welding process parameters.

In this paper, the microstructure characteristics and fracture behavior of steel and aluminum welded joints with and without nickel interlayer under different welding parameters are studied in detail. The influence of nickel interlayer on the organization of the welded joints, as well as the mechanical properties, is discussed, and the metallurgical reaction process during welding is analyzed. The effect of the addition of a nickel intermediate layer on the formation of IMCs was studied, and the strengthening mechanism of the joint was explained.

## 2. Material and Methods

### 2.1. Material

In this paper, dissimilar materials for laser welding of stainless steel–aluminum alloy were investigated, with 6082-T2 aluminum alloy with a thickness of 2 mm, and SUS301L-DLT stainless steel with a thickness of 1.5 mm. Table 1 and Table 2 show the chemical compositions of the two materials, respectively [20,21]. To reduce the generation of brittle and hard aluminum–iron compounds (IMCs), the addition of an intermediate transition layer was used. Nickel foil with 0.05 mm thickness was used as the experimental material. Table 3 shows the main physical properties of the nickel foil intermediate layer.

### 2.2. Material Model

The laser used in the experiment is a TRUMPF TruDisk 4002 disk solid-state laser, assembled with a KUKA robot system to realize automated production with a rated law of 4 KW. The maximum welding speed is 10.5 m/min, the laser beam wavelength is 1.06 μm, the fiber diameter is 0.6 mm, the focal length is 200 mm, the diameter of the compression wheel is 200 mm, and the pressure is 50; through the actual production experience, it is verified in the literature that the welding protective atmosphere has no significant effect on the organization and properties of austenitic stainless steel laser welding 50, and the amount of defocus is 0 mm; through the actual production experience and in the literature, it is verified that the protective atmosphere on the austenitic stainless steel laser welding organization and properties has no significant impact. Therefore, in the production of stainless steel welding using dry compressed air as a protective gas, the gas flow rate is 30 L/min.

**Figure 1:**
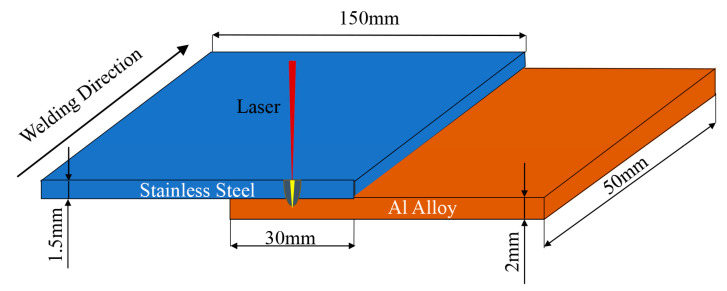
Schematic diagram of aluminum–steel laser deep fusion welding specimen. Experimental results and analysis.

**Figure 2:**
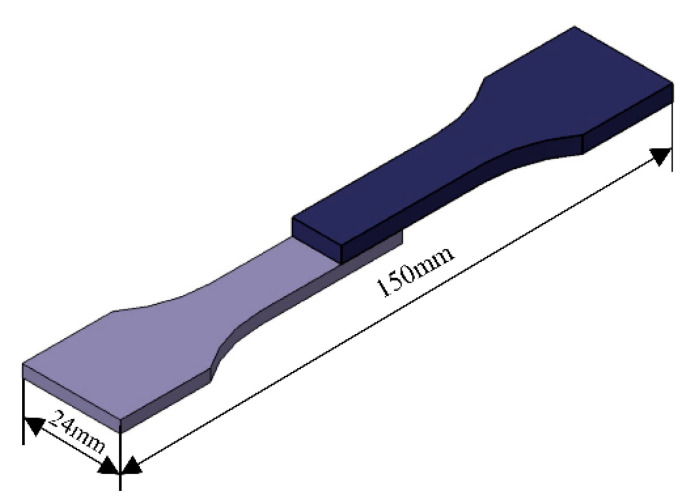
Dimensions of the tensile specimen (mm).

Before laser welding, the upper and lower surfaces of the specimen were polished with sandpaper to remove oxides and oil on the surface of the specimen, and cleaned with acetone. The size of the test plate is 150 mm × 50 mm × 2 mm, stainless steel is on the top, aluminum alloy is on the bottom, and the lap amount is 30 mm. Figure 1 is a schematic diagram of the lap specimen of laser deep fusion welding. In this paper, we use line energy to express the heat input of welding, and the formula of line energy is the ratio of laser power and welding speed. After welding, a ZEISS ScopeA1 optical microscope and Jeol Jsm-IT300 scanning electron microscope were used to study the macroscopic and microscopic organization and morphology of the aluminum/steel joints, and an MH-3 microhardness tester was used to measure the Vickers hardness of each joint. Then, the tensile test was carried out by an MTS-810 electro-hydraulic servo-tester to obtain the tensile strength of joints with different parameters for comparison of the mechanical properties and the effect of adding an intermediate layer on the strength of welded joints. A schematic diagram of the tensile sample is shown in Figure 2.

## 3. Results and Discussion

### 3.1. Surface Quality and Macro-Analysis

Table 4 shows the surface macroscopic morphology of steel/aluminum welds with different line energy for the welding. Samples 1–5 are welds without interlayer, and samples 6–10 are welds with the addition of a 0.05 mm nickel foil interlayer. It can be seen from the table that the weld appearance is well formed, the melting width of the weld surface is small, and no visible cracks are found. As can be seen from the figures, when the welding line energy is small, the weld surface has a bright white metallic luster, and the weld surface is raised and discontinuous. This is mainly due to the excessive oxidation of the surface metal caused by the extreme heat input.

**Figure 3:**
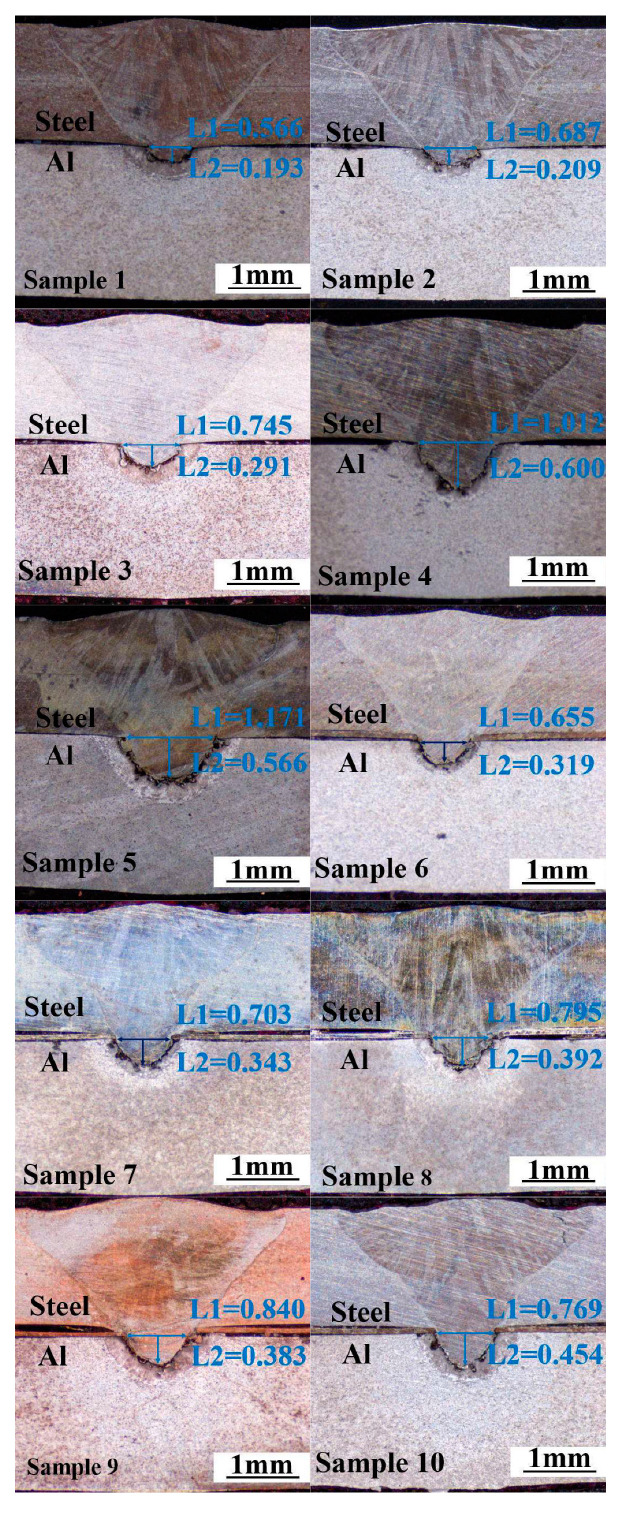
The macro-morphology of the cross-section of the welds with different welding parameters.

Figure 3 shows the macro-morphology of the cross-section of the weld samples 1–10. Under the action of the laser hole effect, the upper plate of stainless steel is melting and embedded in the following partially melted aluminum alloy matrix. The weld profile of the stainless steel part of the upper plate is relatively flat, and it is believed that no intermetallic compounds are generated in this area. However, the lower convex weld enters the lower aluminum alloy plate, and a steel and aluminum reaction zone with a different morphology is formed around the embedded stainless steel in the aluminum alloy lower plate. Some isolated granular and dendritic microstructures can be observed in the fusion zone, indicating that the aluminum alloy and steel react in the fusion zone, and intermetallic compounds are produced.

To study the influence of laser parameters on the macroscopic morphology of the weld, the depth of the molten stainless steel embedded in the lower aluminum alloy plate is used as the depth of the molten pool, and the width of the weld at the junction of the two plates is used as the width of the molten pool. The depth and width of the weld molten pool under different heat input conditions are measured (as seen in Figure 3). It can be seen from the statistics from sample 1 to sample 5 that the penetration depth and penetration width of the welds without the intermediate layer gradually increased with the increase of welding line energy, and the penetration depth increased from 0.193 mm to 0.566 mm. However, when the line energy grew to 113 J/mm, the shape of the weld changed from a “V-shaped” weld to a “nail-shaped” weld, the increase of the weld width was not significant, and the necking phenomenon appeared on the steel–aluminum junction surface. For the welds with nickel interlayer, the variation law of penetration depth and width is the same as without interlayer welds. Still, when the line energy is enormous (114 J/mm), the neck shrinkage phenomenon of the weld is more serious, and the weld width decreases.

It can be seen from the figure that, under the same welding heat input condition, the penetration depth and width of the welds with a nickel intermediate layer are higher than those without the intermediate layer, indicating that the addition of nickel increases the volume of the aluminum alloy pool. When the welding line energy is too large (sample 5 and sample 10), obvious welding cracks will appear on the stainless steel side of the weld, which will lead to a decrease in the strength of the weld.

### 3.2. Microstructure and Phase Analysis

**Figure 4:**
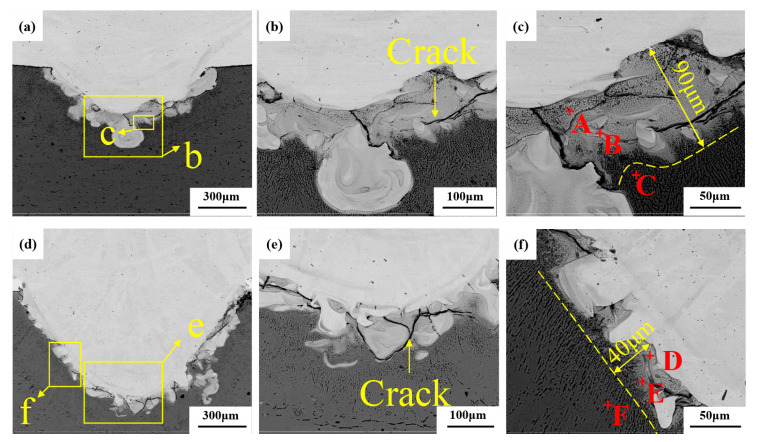
Microstructure of steel-aluminum welded joint (welding line energy =110 J/mm): (**a**) morphology of weld without adding intermediate layers; (**b**) the enlarged morphology of the bottom area of the weld in Figure (**a**); (**c**) the enlarged morphology of the region c in Figure (**a**); (**d**) morphology of weld with nickel intermediate layer; (**e**) the enlarged morphology of the bottom area of the weld in Figure (**d**); (**f**) enlarged morphology of region f in Figure (**d**).

Figure 4 shows the SEM images of welded joints with the welding heat input of 110 J/mm. Figure 4a,d show the overall morphology of the two joints. It can be seen that the molten part of the steel is embedded into the aluminum alloy matrix, and a mixed zone of steel and aluminum is formed on the outside of the embedded stainless steel, indicating that interfacial severe reactions occur between aluminum alloy and stainless steel in the fusion zone. By comparing Figure 4a,d, it is found that the volume of the aluminum alloy molten pool at the bottom of the weld increases with the addition of a nickel intermediate layer under the same welding heat input condition. The melting width of the steel–aluminum bonding surface increases from 0.745 mm to 0.795 mm, and the melting depth increases from 0.291 mm to 0.392 mm. The increase in melting width is also conducive to the improvement of the tensile properties of the joint. Due to the addition of nickel, the diffusion of aluminum is promoted, resulting in a significant increase in weld penetration.

Figure 4b shows the enlarged morphology of the lower part of the steel–aluminum joint. A large number of intermetallic compounds can be seen, and an intermetallic compound layer is formed at the bottom of the weld pool. The reason for the formation of cracks is that the solidification temperature of a large number of intermetallic compounds generated by the reaction is lower than that of the weld metal. In this way, the solidified welded metal first pushes the impurities with a low melting point to the grain boundary of the solidified crystal, forming a liquid film. Moreover, because the cooling rate of the weld pool is very high, the weld metal shrinks during the cooling process. Tensile stress is generated in the weld metal, and this pulls the solidified weld metal apart along the grain boundary. When there is not enough liquid metal as supplement, a tiny crack will be formed. As the temperature continues to drop, the tensile stress increases, and the crack expands. Comparing Figure 4b,e, it can be seen that the presence of nickel affects crack development when adding the nickel intermediate layer, which is conducive to improving the tensile properties of the weld.

**Figure 5:**
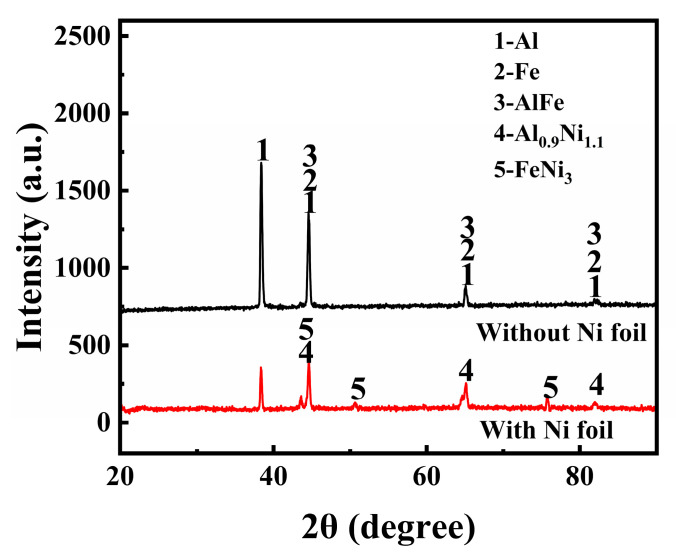
XRD pattern of the welds with/without nickel interlayer.

As can be seen from the enlarged morphology of the intermetallic compound layer in Figure 4c, the layer of the steel–aluminum reaction zone is mainly composed of some acicular intermetallic compounds and some gray solute bands and massive regions. It is speculated that the layer is primarily an Fe-Al intermetallic compound, and the composition of the compound layer is different at different positions of the interface. According to the composition, the compounds can be divided into two categories: one is the iron-rich intermetallic compounds near the steel side with a higher content of iron atoms (FeAl, Fe_3_Al, etc.), and the other is the gray Al-rich intermetallic compounds near the aluminum alloy side with a higher content of aluminum atoms (FeAl_2_, Fe_2_Al_3_, Fe_2_Al_5_, FeAl_3_, etc.). To determine the phase composition of IMCs in the mixed zone of steel and aluminum, EDS and XRD were performed. Table 5 shows the EDS analysis results for welds. The XRD analysis results of the cross sections of the two welds are shown in Figure 5.

It can be seen from Table 5 that the distribution of Fe and Al elements in the intermetallic compound layer is very uneven. Some needle-like structures with high iron can be found in position A, which is close to the steel side. According to the steel and aluminum binary phase diagram and XRD analysis results, the structures in position A are mainly FeAl and FeAl_3_ phases. The location of point B is close to the aluminum side, and the microstructure is a fine strip structure, where the aluminum content is higher and the iron content is lower. According to the composition analysis, the microstructure in this area is mainly Al and FeAl_3_. The composition of the C region is primarily aluminum, which is an Al matrix.

Figure 4d shows the enlarged morphology of the intermetallic compound layer in the weld with nickel interlayer. It can be seen that the thickness of the intermetallic compound layer in the weld with a nickel interlayer is significantly reduced, and the structure is finer than that of the weld without a nickel layer. The EDS scanning results show that there is a high nickel region in the intermetallic compound layer. The content of Fe, Al, and Ni elements in region D is 58.17%, 19.08%, and 7.85%. According to the phase diagram and the XRD results, the central organization of this region is Fe_3_Al + Al_0.9_Ni_1.1_ + FeNi_3_. The content of Al increases, and the content of Fe decreases in position E, which is FeAl + Al_0.9_Ni_1.1_. The position of point F is the aluminum base material, and the content of Fe and Ni lowers until it can be ignored. The addition of Ni significantly reduces the thickness of the intermetallic compound layer from 90 μm to 40 μm.

The intensity of the XRD diffraction peak also shows that the content of brittle Al-Fe compound is less in the weld with nickel interlayer, and the reaction of Ni with Fe and Al produces Fe-Al compound and Fe-Ni compound, so the addition of nickel interlayer has a positive effect on the improvement of mechanical properties of the weld.

**Figure 6:**
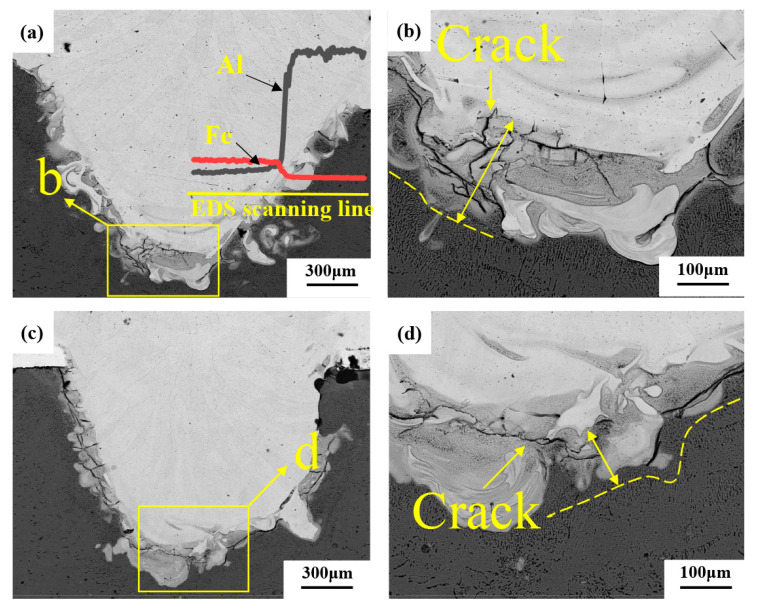
Microscopic morphologies of steel aluminum welded joints (welding heat input is 113 J/mm): (**a**) Morphology of weld without adding intermediate layers; (**b**) The enlarged morphology of the bottom area of the weld in Figure (**a**); (**c**) Morphology of weld with nickel intermediate layer; (**d**) The enlarged morphology of the bottom area of the weld in Figure (**c**).

Figure 6 exhibits the microstructures of the welded joints with the welding heat input of 113 J/mm. As compared to the welded joint without a Ni-foil interlayer, the microstructural morphologies of the weld with Ni interlayer seem to be unchanged, but the molten pool of Al alloy appears to be expanded, as shown in Figure 6a,c. However, the thickness of the intermetallic compound layer is significantly reduced. To study the distribution of elements in the weld, EDS linear scanning was performed, as shown in Figure 6a. The content changes of Fe and Al elements in the interface reaction zone are continuous, and a certain number of Al elements are solidly dissolved in the weld near the stainless steel side. The fluctuation of Fe content is slight, but the content decreases along the scanning direction, which indicates the diffusion of iron into the aluminum matrix, suggesting the formation of the Fe-Al compound in the interface reaction zone. The content of Fe elements fluctuates slightly, but the content decreases along the scanning direction, which indicates that Fe elements diffuse into the aluminum matrix, indicating the formation of Fe-Al compounds in the interface reaction zone.

It was found that several cracks appear in the area without Ni-foil and have a complex microstructure, as shown in Figure 6b. However, only a few cracks were present in the joint with Ni-foil, even at problematic microstructural zones, as shown in Figure 6d. This phenomenon indicates that the welded joint with Ni-foil has a more vital ability to suppress the initiation of the cracks induced by the thermal expansion mismatch than joints without Ni-foil.

### 3.3. Microhardness

**Figure 7:**
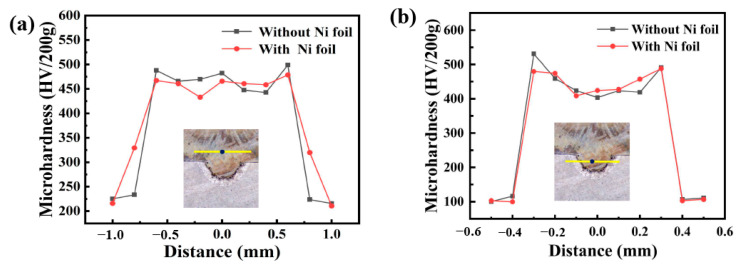
Microhardness profile of the welds with/without nickel interlayer. (**a**) Stainless steel side. (**b**) Aluminum side.

Vickers microhardness measurements with a 200 g loading force and 10 s loading time were carried out on the different locations of the two welded welds, and the measurement results are shown in Figure 7. The yellow lines in the microstructure map indicate the location of the microhardness test, The black dot on the line indicates the starting position for hardness measurement. Figure 7a shows the hardness test results on the bottom of the weld area embedded on one side of the aluminum plate, and it can be seen that the hardness of the liquation zone outside the embedded area of the aluminum alloy is significantly higher than that of the base metal and fusion zone. This is because, during the process of laser welding, Al enters into the Fe lattice to form α-Fe solid solution due to the stirring effect of the molten pool and generates brittle IMCs. The maximum hardness of the weld without the intermediate layer reaches 531 HV, which is significantly higher than that of the weld with the nickel intermediate layer. EDS analysis results of the weld area also proved and, due to the addition of Ni interlayer, intermetallic compounds of FeNi_3_ and Al_0.9_Ni_1.1_ were generated in the fusion area, which prevented part of the reaction of aluminum and iron, reduced the formation of brittle and hard FeAl_3_ and FeAl phases, and reduced the average hardness value of the weld, which was conducive to improving its mechanical properties.

Figure 7b shows the microhardness test results of the upper steel side of the weld. It can be seen that the hardness of the welded joint is significantly higher than that of the base material (200 HV) on the steel side. Still, the hardness distribution in the fusion zone is uniform, and the average hardness is about 460 HV, which is mainly due to the quenching effect of the steel. In the thermal influence zone (HAZ), the hardness of the steel is increased (350 HV), which may be related to the fine needle organization caused by grain refinement and quenching during recrystallization.

### 3.4. Tensile Properties

**Figure 8:**
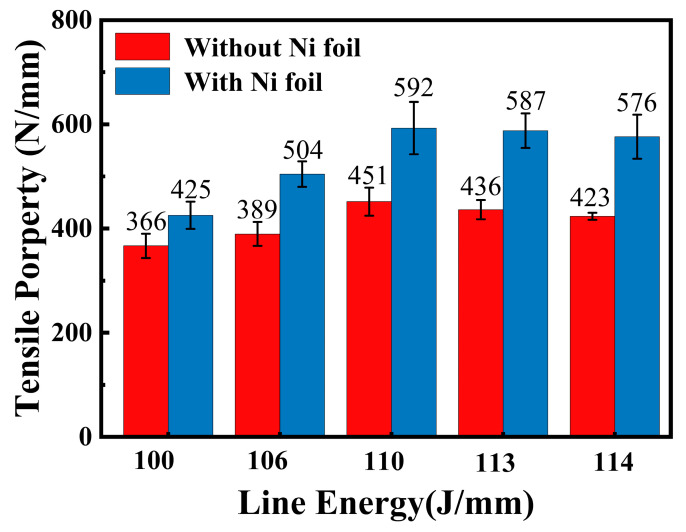
Tensile property of the welds with/without nickel interlayer.

Tensile shear experiments were carried out on the welded specimens, the dimensions of which are shown in Figure 8. As can be seen, the line energy has a significant effect on the tensile properties of the welded joints. With the increase of welding line energy, the tensile shear strength of the weld without and with the nickel interlayer increases first and then decreases. For the weld without an intermediate layer, the highest tensile shear strength is 592 N/mm when the laser welding energy is 113 J/mm. After that, the line energy continues to increase, but the mechanical properties of the welded joint decrease. For the weld with nickel interlayer, when the welding energy is 110 J/mm, the tensile shear is maximum (451 N/mm). As the line energy continues to increase, the welding heat also increases, which makes it easy to form spatter in the welding process, resulting in weld surface depression. Although the weld width increases, the higher energy input leads to the metallurgical reaction of aluminum alloy and stainless steel, forming a large number of brittle aluminum and iron compounds, and the welded joint is prone to fracture, which leads to a significant decline in the strength of the weld. Therefore, for steel and aluminum joints without and with Ni intermediate layer, the best welding line energy is 110 J/mm.

Under the same welding line energy condition, the tensile shear strength of steel and aluminum welding joints with a nickel intermediate layer is higher than that without an intermediate layer. When the line energy is 110 J/mm–113 J/mm, the tensile shear strength of the samples with nickel interlayer is more than 580 N/mm, which is 43% higher than that of the weld without the intermediate layer. From the results of the tensile tests of the welds in Figure 8, the Ni interlayer can increase the tensile properties of the joint, besides increasing the width of the jointing zone due to the expansion of the molten pool of Al alloy, due to the improvement of the toughness of the joint with an Ni interlayer.

### 3.5. Fracture Morphology

**Figure 9:**
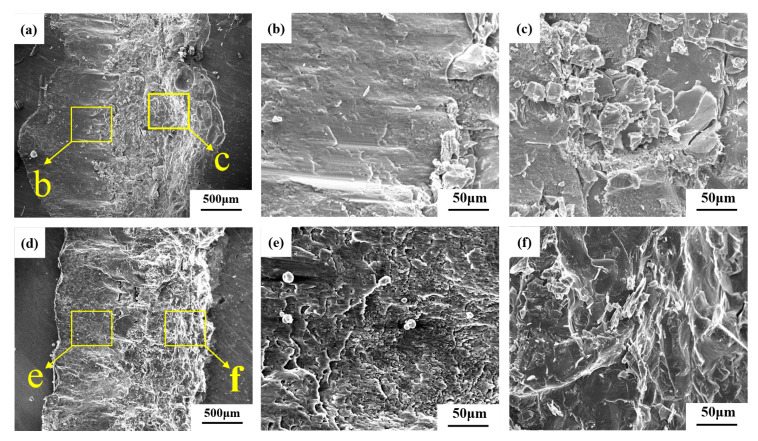
Fracture morphologies of the joints (welding heat input is 110 J/mm).

Figure 9 shows the fracture morphology of the stainless steel side of tensile specimens of two kinds of welds when the welding line energy is 110 J/mm, in which Figure 9a–c is the fracture SEM morphology of the weld without an intermediate layer and Figure 9d–f is the fracture SEM morphology of the weld with nickel intermediate layer. The weld fracture occurs at the intermetallic compound of the bonding surface of stainless steel and aluminum alloy due to the heterogeneity of the joint, which is mainly caused by the brittle and hard phase generated in the fusion zone of steel and aluminum. As can be seen from the overall morphology of the two tensile fractures in Figure 9a,c, the entire fracture surface is divided into two regions: the slip separation zone and the ductile/brittle fracture zone. Although the joints with and without Ni interlayer have the same fracture modes, the fracture surfaces are different. The section of the slip separation area without the tensile fracture of the weld in the middle layer is relatively flat, and flake slip bands appear in the enlarged figure in Figure 9b. Figure 9c shows the enlarged morphology of the fracture zone of the weld without the middle layer. It can be seen that the stepped tear edges and the fine cracks distributed along the grain boundaries show obvious brittle fractures. Figure 9d shows the fracture morphology of the weld with the nickel intermediate layer, which presents the characteristics of plastic fracture and inclusion of a brittle fracture region. From the enlarged morphology of the slip separation region in Figure 9e, it can be seen that there are local dimples due to plastic deformation. The joint with a Ni foil has roughly higher toughness than the joint without a Ni foil because it has a larger fracture surface with the base metal. In Figure 9f, the enlarged morphology of the fracture area can be shown as a river-like pattern, showing some ductile fracture characteristics. Compared to the weld without Ni interlayer, the cracks decrease significantly, which means that the joint with the Ni-interlayer has crack resistance.

## 4. Conclusions

In this article, laser welding of the steel-on-aluminum overlap configuration with and without Ni interlayer were investigated. The main conclusions of this paper are as follows:

1. During laser welding, the melting of stainless steel embedded into the aluminum matrix and an interfacial metallurgical reaction with the molten aluminum alloy have occurred to form an intermetallic compound layer. The structures of the intermetallic compound layer without Ni interlayer consist of FeAl and FeAl_3_. After the addition of the nickel intermediate layer, the thickness of the intermetallic compound layer and the content of the brittle and hard Al-Fe phase decreased significantly, and some new phases of Al_0.9_Ni_1.1_ and FeNi were formed.
2. The tensile properties of the joints with and without Ni interlayer first increase and then decrease with increase in the line energy. Using the nickel interlayer can expand the volume of the aluminum melted pool, enhanced the tensile property of the joint by about 40% and decrease the microhardness of the intermetallic compounds.
3. Improving the metallurgical reaction of the molten pool for laser welding by adding a Ni interlayer is an effective way of enhancing the mechanical properties of the joint. Using the Ni interlayer can significantly reduce the thickness of the IMCs layer, and change the structure of the IMCs layer. The fracture morphology of the joint changes from obvious brittle fracture to partial toughness fracture, indicating the enhancement of the toughness of the joint and the tensile properties. In subsequent experiments, we would like to improve the effect of different thicknesses of nickel foil on welded joints.

## Figures and Tables

**Table 1:** Chemical composition of SUS301L austenitic stainless steel (wt%).

C	Si	Mn	P	S	Ni	Cr	N	Fe
0.03	1.0	2.0	0.045	0.03	7.0	17.0	0.1	Bal.

**Table 2:** Chemical composition of 6082 aluminum alloy (wt%).

Mg	Si	Cu	Fe	Mn	Cr	Zn	Ti	Al
0.97	1.2	0.01	0.22	0.87	0.18	0.02	0.04	Bal.

**Table 3:** Main physical properties of interlayer materials.

Metal Name	Density/g·cm^−3^	Melting Point/°C	Specific Heat /J·kg^−1^·K^−1^	Thermal Conductivity/W·m^−1·^K^−1^	Linear Expansion /10^−6^·K^−1^	Resistivity/10^−6^Ω·m
Nickel	8.90	1453	240	90.7	5.2	6.84

**Table 4:** Surface morphology of welds with different welding process parameters.

Test Piece Number	Line Energy (J/mm)	Intermediate Layer	Macrostructure
Sample 1	100	No	** 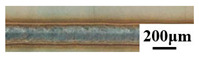 **
Sample 6	100	0.05 mm Ni	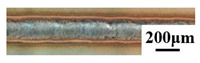
Sample 2	106	No	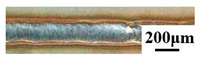
Sample 7	106	0.05 mm Ni	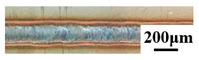
Sample 3	110	No	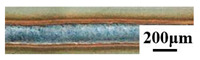
Sample 8	110	0.05 mm Ni	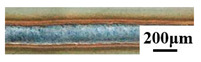
Sample 4	113	No	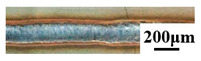
Sample 9	113	0.05 mm Ni	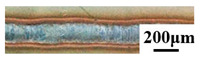
Sample 5	114	No	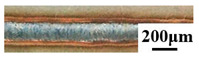
Sample 10	114	0.05 mm Ni	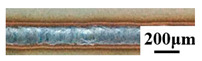

**Table 5:** EDS Scanning Results.

Region	Composition%						Potential Phases
	Al	Fe	Cr	Si	Ni	Mn	
A	14.28	59.49	18.02	0.91	5.81	1.48	Fe_3_Al + FeAl
B	96.56	1.32	0.39	1.08	0.13	0.12	Al + FeAl
C	98.72	0	0	1.25	0	0.03	Al
D	19.08	58.17	13.93	0.88	7.85	0.13	FeNi_3_ + Al_0.9_Ni_1.1_
E	55.77	31.26	7.91	1.22	3.47	0.37	FeAl + Al_0.9_Ni_1.1_
F	97.29	0.57	0.62	0.78	0.06	0.14	Al

## Data Availability

Datasets generated and/or analyzed during the current study are available from the corresponding author on request.
